# New Insights into Monoclonal B-Cell Lymphocytosis

**DOI:** 10.1155/2014/258917

**Published:** 2014-09-11

**Authors:** Christina Kalpadakis, Gerassimos A. Pangalis, Sotirios Sachanas, Theodoros P. Vassilakopoulos, Stavroula Kyriakaki, Penelope Korkolopoulou, Efstathios Koulieris, Maria Moschogiannis, Xanthi Yiakoumis, Pantelis Tsirkinidis, Marie-Christine Kyrtsonis, Georgia Levidou, Helen A. Papadaki, Panayiotis Panayiotidis, Maria K. Angelopoulou

**Affiliations:** ^1^Department of Haematology, University Hospital, University of Crete, P.O. BOX 1352, 71110 Heraklion, Crete, Greece; ^2^Department of Haematology, Athens Medical Center-Psychikon Branch, 11525 Athens, Greece; ^3^Department of Haematology, University of Athens, Laikon General Hospital, 11527 Athens, Greece; ^4^Department of Pathology, University of Athens, 11527 Athens, Greece; ^5^Department of Haematology, 401 Military Hospital, 11525 Athens, Greece; ^6^1st Department of Propedeutics, University of Athens, Laikon General Hospital, 11527 Athens, Greece

## Abstract

Monoclonal B-cell lymphocytosis (MBL) is a premalignant condition characterized by the presence of less than 5000/*μ*L circulating clonal B cells in otherwise healthy individuals. Three subcategories have been identified according to the immunophenotypic features: CLL-like, CD5(+) atypical, and CD5(−) MBL. CLL-like MBL is by far the most frequent and best studied category and further divided in low-count [LC] and high-count [HC] MBL, based on a cutoff value of 500/*μ*L clonal B cells. LC-MBL typically remains stable and probably does not represent a truly premalignant condition, but rather an age-related immune senescence. On the other hand, HC-MBL is closely related to CLL-Rai0, bearing similar immunogenetic profile, and is associated with an annual risk of progression to CLL requiring therapy at a rate of 1.1%. Currently there are no reproducible factors for evaluating the risk of progression to CLL. CD5(−) MBL is characterized by an immunophenotype consistent with marginal zone origin and displays many similarities with marginal zone lymphomas (MZL), mainly the splenic MZL. The cutoff value of 5000/*μ*L clonal B cells cannot probably be applied in CD5(−) MBL, requiring a new definition to describe those cases.

## 1. Introduction 

Multiparameter flow cytometry analysis dramatically increased the sensitivity for detection of small B-cell clones in otherwise healthy individuals [1–7]. A variety of terms have entered the literature to designate this finding. In 2005 the International Familial CLL consortium summarized the literature, proposed certain diagnostic criteria to define this entity, and established the term “monoclonal B-cell lymphocytosis” (MBL) [8]. MBL is characterized by an asymptomatic monoclonal expansion of <5.0 × 10^9^/L circulating B cells in apparently healthy individuals without any other feature diagnostic of a B-lymphoproliferative disorder. MBL is classified into three groups according to the immunophenotype of the clonal population. The majority of MBL cases (75%) have the immunophenotype of chronic lymphocytic leukemia (CD19(+), CD5(+), CD23(+), CD20^dim⁡^, and sIg^dim⁡^) and are classified as typical CLL-like MBL, while the remaining MBL cases are classified as atypical CLL (CD5(+) clonal population not meeting the criteria for typical CLL and not meeting the criteria for mantle cell lymphoma) and as CD5(−) non-CLL MBL [4, 5, 8].

## 2. CLL-Like MBL

### 2.1. Prevalence

The reported prevalence of MBL ranges widely from <1% to more than 18%, depending on the sensitivity of flow cytometry and the populations tested (Table 1). The absence of standardized flow cytometry methods for MBL diagnosis complicates determination of the true prevalence. Early population studies indicated an MBL prevalence of 0.1% to 3% by screening for B-cell clones with 1- to 3-color flow cytometric analysis [9, 10]. Studies using 4- to 5-color protocols revealed a higher prevalence, ranging from 3% to more than 6% [4, 5, 12]. In the most sensitive study published to date, Nieto et al. utilized the highest sensitivity flow cytometry approaches available with the use of 8-color staining panels and the analysis of 5 million B cells per subject and identified an MBL prevalence of 14% among 608 healthy adults aged more than 40 years [3]. So, as the sensitivity of flow cytometry increases by the use of multicolor techniques and the analysis of greater numbers of B cells per subject, the prevalence of MBL increases over time.

MBL prevalence also varies based on the studied population. In the general population studies MBL is detectable in approximately 4% to 5% of adults when typical flow cytometric techniques are used (4-color with a detection sensitivity of 1 : 10000 events) [4, 5, 12]. General trends across studies indicate a greater risk of MBL among those with increasing age and among men, similar to CLL [4–6]. In the Spanish study, a CLL-like MBL was detected in more than 1 in 5 individuals over 60 years old [3, 7]. Furthermore, the same group, based on the analysis of 638 healthy adults (>44 years old), investigated the association between the frequency of CLL-like MBL and the volume of sample analyzed. Based on this model, they suggested that the frequency of MBL cases would rise to 100% for subjects older than 70 years when more than 45 mL of peripheral blood was analyzed [7]. In a recent study in 2098 healthy blood donors with the use of 6-color flow cytometry and the analysis of at least 500000 events yielded an overall prevalence of 7.1% [13]. The prevalence was higher in men than in women and increased with age in both genders. There was ~1.4-fold increase for every 10 years in the MBL prevalence among men, starting from 6.0% in the 45–64-year-old group and reaching 12.2% in the 65-year or older group.

MBL is more common in first-degree relatives of patients with familial CLL, with a reported prevalence of 12% to 18% [14–16]. This elevated MBL risk is particularly evident in young adults aged 16 to 40 years with a 17-fold relative risk, suggesting that there is an inherited abnormality that increases susceptibility to development of MBL at a much earlier age than the general population. In relatives of patients with sporadic CLL the overall prevalence has been reported in the range of 4%, similar to that detected in the general population. However, in individuals aged more than 60 years from families with sporadic CLL, the MBL risk seems to be significantly increased (approximately 16%) similar to that seen in relatives of familial CLL cases [14].

In outpatient series the prevalence of MBL is almost the same as in the general population as long as patients with normal blood counts are encountered [4, 5]. However in one study on 2228 outpatients aged 39–99 years who were referred for investigation of lymphocytosis, the prevalence of MBL was much higher, in the range of 13.9% [11].

### 2.2. Classification of MBL into Low and High Count

In the general population studies the majority of MBL cases have very low numbers of clonal B cells, typically in the range of 0.1–10/*μ*L, with a median number of clonal B cells 0.001 × 10^9^/L [20]. In contrast, MBL cases identified after investigation of absolute lymphocytosis typically have clonal B-cell counts above 450/*μ*L while the median absolute clonal B-cells count is 2,939/*μ*L [11, 18].

Based on this heterogeneity of the size of the B cell clone, MBL is now subdivided into two categories: high-count MBL (also known as clinical MBL) and low-count MBL [20, 21]. A cutoff value of 0.5 × 10^9^ clonal B cells/L has been proposed by the literature for discriminating LC-MBL and HC-MBL [20, 21]. This cutoff value seems to be of clinical relevance, since the majority of MBL cases carry either very low-count clones (<56 clonal B cells/*μ*L) or more than 1500/*μ*L clonal B cells, with different risk of progression [20].

Of note, both low- and high-count MBL cases usually display multiclonality, in contrast to CLL in which multiclonality is much less common [22, 23].

### 2.3. Clinical or High-Count MBL

HC-MBL is closely related to CLL-Rai0, as it has been shown by several series [11, 18, 20, 21, 24]. Current data indicate CLL is more likely to develop in individuals with clinical MBL than in those with low-count MBL [20]. In the study by Rossi et al. 123 HC-MBL cases were compared to 154 CLL Rai stage 0 cases [18]. Demographics were similar. MBL cases were characterized by lower percentage of bone marrow infiltration, better preserved immune function, more favorable genetic profile, slower disease kinetics, and longer TFS compared to CLL-0, suggesting that HC-MBL is a distinct entity. However, another study by Kern et al. compared 298 MBL cases with 356 CLL patients with regard to biological characteristics and cytogenetics and suggested that these two entities largely overlap and represent closely related stages of the same disease that differ only in tumour mass and that the separation of MBL and CLL by a threshold of 5,000/*μ*L clonal B cells cannot be reproduced by genetic aspects [25]. Studies including cases with clinical MBL have shown that the annual risk of progression to CLL requiring therapy is 1% to 2% (Table 2) [11, 17, 26, 27]. In the Leeds study 185 CLL-like MBL cases were followed for a median of 80 months and 1.1% per year required treatment for disease progression [20]. Similar results were observed in the Mayo Clinic studies which reported on 302 CLL-like MBL cases and observed a 1.4% annual risk of progression to CLL requiring therapy with a median follow-up time of 18 months [17]. In the Italian study, 123 clinical CLL-like MBL cases were monitored for a median of 43 months and it was observed that 4% per year required therapy in the first 7 years and decreased thereafter to 0% [18]. These studies also showed that the absolute B-cell count is the most important determinant of progression to CLL. However there is not any established cutoff point of B-ALC to predict the risk of progression to CLL (Table 2). In the Italian study, a B-ALC greater than 3.7 × 10^9^ B cells/L predicted the highest risk of developing CLL or SLL, whereas a B-ALC at presentation below 1,200/L was the optimal cutoff point to predict a stable lymphocyte count [18]. In the UK study cutoff points were similar to the Italian one [20]. A B-ALC below 1,200/L and above 4,000/L was the optimal cutoff point for predicting a stable and a rising lymphocyte count, respectively [20]. A Mayo Clinic study by Shanafelt et al. included patients with MBL and Rai stage 0 CLL and observed that a threshold of 11,000/L B-ALC at diagnosis optimally predicted treatment-free survival as well as overall survival [28]. A similar cutoff point of 10,000/L B-ALC has been reported by Molica et al. as well as by Scarfo et al. as a predictor for time to first therapy [19, 29, 30]. In the latter study, by using a cutoff level of 10,374/*μ*L clonal B cells, the difference in terms of annual risk of progression became larger (2.4 versus 8.5% per year) compared with the difference based on the traditional cutoff level of 5,000/*μ*L clonal B cells (1.1% versus 5.2% per year) [19].

The above data indicate that, by using a higher cutoff level of B-ALC instead of the currently used (5,000/*μ*L) for discriminating MBL from CLL Rai stage 0, it seems that it can better assess the risk of progression. However studies are ongoing investigating the best cutoff value for discriminating MBL into LC-MBL and HC-MBL, with distinct biological and clinical significance, as well as for discriminating HC-MBL from CLL Rai 0. Given the fact that B-cell count is a continuous variable, it is rather impossible to establish any specific B-cell count threshold to precisely identify MBL cases with no risk of progression [19].

Assessing the B-cell count seems not to be enough to sharply demarcate the lowest and the highest risk categories. Biological parameters may be needed to best stratify patients [19]. Several factors have been assessed for predicting outcome of MBL, but with conflicting results. Biological factors that have been proposed include IGHV homology, CD38, CD49d, and ZAP-70 expression, and FISH karyotype [18, 20, 21, 28]. Cytogenetic parameters as well as mutation status seem to have the best prognostic power for predicting the risk for progression to CLL [18, 25].

Another important finding of these studies is that progression to CLL did not plateau over a long follow-up time, indicating that clinical MBL always progresses to CLL if given sufficient time [31]. However, only a small number of patients will actually experience disease progression due to the low progression rates per year and the age of subjects.

Besides the risk for progression to CLL requiring therapy, HC-MBL carries also a higher risk of serious infection than the general population. Based on the Mayo Clinic database, Moreira et al. reported that HC-MBL is at ~3-fold higher risk of hospitalization with infection compared with a control population and that this risk is fourfold greater than the risk for progression to CLL requiring therapy [32]. Interestingly, this increased susceptibility to infections was not associated with hypogammaglobulinemia.

### 2.4. Low-Count or Population Screening MBL

Low-count MBL can be detected only by applying highly sensitive flow cytometry techniques in otherwise healthy individuals. Data on the outcome of low-count MBL is limited. Fazi et al. reported on 76 patients with LC-MBL and observed that, after a median follow-up time of 34 months, 90% of the cases persisted over time in contrast to only 44.4% and 66.7% of atypical CLL and CD5(−) MBL, respectively [33]. Furthermore they reported that most of the LC-MBLs remained stable without progression to clinically overt disease, suggesting that the potential risk of progression into overt CLL is exceedingly rare and definitely less than that of clinical MBL. These findings confirm the hypothesis that the natural history of LC-MBL differs from that of HC-MBL and LC-MBL does not represent a true preleukemic condition, in contrast to HC-MBL. These results are in line with the molecular and biologic differences observed between the two subgroups of MBL [12, 34, 35].

Since the prevalence of LC-MBL is much higher in the elderly population with a peak of 75% in persons above 90 years of age, it seems that LC-MBL may represent an epiphenomenon of immunosenescence, observed in the elderly [33, 34]. In accordance with this hypothesis is the finding of increased clonal T-cell populations [33]. In more than one-half of the cases multiple T-cell clones were identified, compared with the general population suggesting a widespread deregulation of the immune system in MBL. Clonal expansions of T cells are frequently observed in elderly individuals [36]. Moreover, it has been shown that LC-MBL is associated with reduced numbers of normal B-cell subsets, mainly of immature and naïve B cells [37].

### 2.5. Bone Marrow Histopathology

Bone marrow examination is not required for the establishment of MBL diagnosis. Therefore there is limited data on bone marrow findings in MBL [38]. Our group has recently evaluated the histopathological and immunohistochemical findings of bone marrow biopsies (BMB) in a series of 48 cases (data unpublished). The median percentage of bone marrow infiltration was 28% (range, 5–85%). The pattern of infiltration was interstitial or mixed (nodular and interstitial) in the majority of the cases, 88%. There was no correlation between the extent of BM infiltration and the absolute number of peripheral blood monoclonal B cells.

### 2.6. Cytogenetic and Molecular Features of MBL

Significant progress has been made recently regarding the molecular and cytogenetic aspects of MBL. Reports of chromosomal abnormalities in CLL-type MBL indicate that both LC-MBL and HC-MBL carry the same cytogenetic aberrations associated with good prognosis CLL [3, 11, 17, 18, 25, 35, 39, 40]. The frequency of 13q deletions, the most favourable cytogenetic subgroup in CLL, is similar to that observed in newly diagnosed CLL, since it has been detected in more than one-third of CLL-like MBL, in both subcategories (low and high count). Even in the Salamanca series, del13q was detected in 36% of the cases, whereas in the Italian series by Fazi et al. del13q was evident in 43.8% of the LC-MBL cases [3, 33]. This suggests that 13q deletion occurs early in the natural history of CLL-like MBL and is probably not associated with the disease progression [41]. A similar finding is seen for trisomy 12 which has been detected in 8–22% of cases among different series including both HC-MBL and LC-MBL [3, 11, 17, 25, 33, 35]. However deletions of 11q and 17p, which are seen in CLL in association with a poor prognosis, are infrequent in HC-MBL and LC-MBL [3, 11, 17, 25, 33, 35]. Overall, LC-MBL shows a lower frequency of genetic alterations associated with CLL than HC-MBL and CLL. Furthermore, coexistence of ≥2 cytogenetic alterations is less frequent in MBLs than in CLL [35].

LC-MBL and HC-MBL have similar somatic hypermutation status, since more than two-thirds of the cases carry somatically mutated IgHV genes [12, 25, 35, 39]. In the study by Vardi et al. [34], unmutated IgHV genes were used in approximately 25% of LC- and HC-MBL cases, similarly with CLL-0, while CLL > 0 cases displayed unmutated IgHV genes in a significantly higher frequency (~45%). Significant differences have been identified regarding the usage of IgHV genes as well as the Ig gene repertoire between LC-MBL and HC-MBL [12, 34, 35]. The IGHV gene repertoire of LC-MBL displays pronounced differences compared with HC-MBL and CLL-0, such as suppressed frequency of the IGHV1-69,* IGHV4–34*, and* IGHV3–23* genes and overrepresentation of the IGHV4–59/61 genes [12, 34]. The latter genes are found with increased frequency in elderly individuals, confirming the notion that LC-MBL may represent an aspect of the age-related immune senescence [42]. In contrast to LC-MBL, the IGHV repertoire of HC-MBL closely resembles that of mutated CLL, since the* IGHV3–07*,* IGHV3–23,* and* IGHV4–34* genes are used in around half of the cases [12, 34]. A recent study by Orfao's group based on 78 CLL-like MBL and 117 CLL clones showed that certain patterns of IGHV gene usage are associated with specific genetic alterations [35]. Based on these findings the authors identified three different groups: a group including mainly LC-MBL commonly expressing the VH3-23 gene with no or isolated good prognosis cytogenetic alterations, another group which mainly consisted of HC-MBL and advanced-stage CLL with a common usage of the VH1-69 gene along with the presence of poor prognosis cytogenetic alterations, and a third group with intermediate features. Another important difference between LC-MBL and HC-MBL is the finding that BcR stereotypy is exceedingly rare in LC-MBL, in contrast to HC-MBL and CLL, in which BcR stereotypy is a distinctive feature being present in almost one-third of patients [34, 43].

All the above findings further support the notion that LC-MBL does not represent a truly preleukemic condition, but more possibly a physiological process of age-related immune senescence, despite being clonal. HC-MBL, on the other hand, displays significant similarities with CLL-0 at both the clinical and the biological level [18, 25]. In the largest series published today including 333 CLL-like MBLs, HC-MBL exhibited the same frequency of unmutated rearrangements (~25%) with CLL-0 [34]. Furthermore, both HC-MBL and CLL-0 cases presented similar IGHV gene repertoire, which differed from that of LC-MBL and CLL > 0. Another important observation from the aforementioned study is the presence of BcR stereotypy in the same frequency as in CLL-0 (~20%), further supporting the possibility that these two entities have common immunogenetic profile.

Most CLL-like MBL cases display an indolent and stable clinical course. However a small proportion of HC-MBL cases will eventually progress to CLL. On the other hand, it has been shown that virtually all CLL are preceded by an MBL [3]. Since only a minority of MBLs will actually progress to CLL requiring therapy, it is of significant importance to be able to discriminate at diagnosis these rare cases which will eventually progress into a malignant disorder. Understanding the key mechanisms involved in the expansion of the MBL clones may help in a better understanding of the natural history of the disease and to modify our strategies for correctly managing B-cell premalignant states. Given the fact that cytogenetic abnormalities are commonly detected even in LC-MBLs, there is a possibility that their role may be very limited or even nonexistent in the early phases of MBL development [34, 41]. The underlying mechanisms responsible for the development and evolution of MBL into CLL are not known yet. It is of interest that LC-MBL remains stable despite the fact that the proliferative stimuli, such as persistent Ag-induced activation of the BCR, lead to the acquisition of genetic aberrations. On the contrary, in HC-MBL there is probably a genetic predisposition leading to quick transit from the LC-MBL to the HC-MBL/CLL phase [34]. Autonomous genetic abnormalities (e.g., a single mutation) affecting the same or parallel critical signaling pathways may further support and amplify the initial clonal expansion [41]. Among these multiple pathophysiologic mechanisms for the progression of MBL to CLL, antigen stimulation seems to play a critical role. Evidence exists in the literature for a link between infections and increased risk to develop CLL [44, 45].

Next generation sequencing technologies have allowed the identification of several genomic alterations in CLL with prognostic significance, such as mutations of ATM, p53, NOTCH1, SF3B1, and BIRC3 genes that might represent new biomarkers of potential clinical relevance [46–48]. In MBL these secondary lesions are found in a significantly lower frequency (0%–3%) than in CLL [23, 49, 50]. However a recent multicenter trial by the Gruppo Italiano Studio Linfomi reported a much higher prevalence of NOTCH 1 mutations in 100 MBL cases (11%) by using a highly sensitive method [51].

Recently it has been proposed that microRNAs (miRs) are involved in the transition from monoclonal B-cell lymphocytosis (MBL) to CLL [52]. In a study, Ferrajoli et al. tested miR-15a/16-1 cluster, miR-21, and miR-155 expression in purified B cells of normal individuals, individuals with MBL, and patients with CLL, found that miR-155 was overexpressed in B cells from individuals with MBL and even more so in B cells from patients with CLL, when compared with B cells from normal individuals, and supported the use of cellular and plasma levels of miR-155 as biomarkers for the risk of progression in individuals with MBL [53].

Several studies have shown the existence of multiclonality in a significant proportion of MBL cases (up to 20%) [22]. A recent study showed that the B-cell receptor of B-cell clones from multiclonal cases presented a slightly higher degree of HCDR3 homology than B-cell clones from monoclonal cases, in association with unique hematological (e.g., lower B-lymphocyte counts) and cytogenetic (e.g., lower frequency of cytogenetically altered clones) features usually related to earlier stages of the disease. Based on these findings, the authors supported the antigen-driven nature of such multiclonal B-cell expansions, with potential involvement of multiple antigens/epitopes [54].

The conclusion from the above studies is that the progression of MBL to CLL probably involves multiple pathophysiologic mechanisms including critical gene mutations and microenvironmental stimulation along with a CLL-prone genetic background [41].

## 3. Practical Aspects Of MBL Diagnosis: Unresolved Issues

### 3.1. Establishment of Reproducible Prognostic Factors: Drawing the Line between MBL and CLL-0

There is active ongoing research in the field of molecular pathogenesis and progression of MBL to CLL. Significant advances have been recorded so far in order to better understand the clinical aspects of this entity. Currently, it is still a matter of debate which are the best B-cell cutoff value and biological or cytogenetic factors for predicting the risk of progression. Further studies are required in order to determine factors with prognostic significance that would allow us to identify, early, MBL cases with high risk for progression to CLL requiring therapy. On the other hand, among CLL-0 cases, a significant proportion would not progress. Ideally, in the future the category of clinical MBL could be applied for all these cases (MBL and CLL-0) with no risk for progression (irrespectively of their MBL count) and stratify into the group of CLL-0 only those cases with a tendency to progress [41].

### 3.2. Staging Procedures

According to the guidelines for MBL evaluation at diagnosis, no imaging studies are required [8]. This raises concerns about the possibility that at least a proportion of cases classified as MBL could in fact represent small lymphocytic lymphoma [55] with a nodal burden not evident by physical examination (e.g., intra-abdominal lymphadenopathy). There is limited data evaluating this topic. In the series by Scarfo et al. the vast majority (155/165) of the HC-MBL cases imaging studies were negative, arguing against this hypothesis [19]. Further studies are required to clarify the role of imaging studies in non-CLL-like MBL.

### 3.3. MBL in Blood Donors

Several of the general population studies for the identification of MBL have been performed in blood donors [10, 13]. Recently, Shim et al. report that MBL is a surprisingly common finding in healthy blood donors in the range of 7.1% [13]. This finding raises concerns regarding the potential risk of transfer of a premalignant condition to recipients of blood transfusion [56]. Given the fact that there is an increased risk for development of a B-cell malignancy, particularly in CLL, with blood transfusions [57], it is very important to clarify this issue. Since LC-MBL is not typically associated with a risk of progression, blood products from such donors are probably acceptable. The potential risk mainly involves blood donors with HC-MBL. So the question is whether donors with mild lymphocytosis should be screened for MBL. In order to answer this question further studies are required. Until then, the recommendation of Shim et al. for a conservative approach to blood transfusions is warranted [13].

### 3.4. MBL in Transplant Donors

The implications for allogeneic transplantation are significantly more complicated than blood donation, especially if the transplant is for a CLL patient. MBL prevalence is especially high in relatives of familial CLL cases reaching 18% in some studies [15]. Given this high incidence, it is probably reasonable to recommend that potential HLA-matched sibling donors for CLL patients be tested for the presence of MBL or early CLL [58]. Currently, there is limited data evaluating the risk of MBL transfer to recipients and the impact on overall survival after transplantation.

### 3.5. Ethical Considerations

Clinicians usually face difficulty on how to inform and follow up an individual with a diagnosis of MBL. The most sensible strategy, for the HC-MBL cases, is to reassure them that MBL is not a malignant entity and that the risk of progression to CLL is low, but not negligible, indicating a yearly hematologic consultation with a complete blood cell count and physical examination [31, 41]. For LC-MBL, which is identified in general population studies after the application of high-sensitivity flow cytometry methods, the risk of progression to CLL is very low, if any. Based on these data, it would be appropriate not to inform individuals for having MBL and not to prompt any monitoring [34, 41].

## 4. Commentary on CLL-Like MBL

Based on the above data the following comments can be made. CLL-like MBL is a quite common condition, being at least 100 times more frequent than CLL. Based on the absolute clonal B-cell count, MBL is further divided into low- and high-count MBL. Not all MBL carry the same risk of clinical progression. The absolute clonal B lymphocyte count is the only well-established prognostic factor so far for the detection of the risk for the progression of MBL into CLL. New data on the biology of MBLs may help to better discriminate the subset of MBLs which are more likely to progress from those cases with no propensity to progression. LC-MBL (<500/*μ*L clonal B cells) is identified in healthy adults during screening-population studies, while clinical or HC-MBL is usually identified during evaluation of lymphocytosis. Based on the current data, LC-MBL is a condition with no clinical relevance and do not require any monitoring. It appears to represent a physiological process of age-related immune senescence rather than a truly premalignant condition. Finally, HC-MBL closely resembles CLL-Rai0 and research is ongoing to identify factors that could help in discriminating those cases with high risk for progression.  Table 3 summarizes the main differences in clinical, cytogenetic, and molecular characteristics between HC-MBL, LC-MBL, CLL-0, and CLL > 0.

## 5. CD5(−) MBL

Data on the clinical aspects and biological significance of non-CLL-like MBL is limited. Non-CLL-like MBL has generally been subdivided into two major groups: CD5(−) MBL and atypical CD5(+) MBL. Atypical MBL displays CD5 positivity along with higher expression of CD20 and other immunophenotypic features different from typical CLL-like MBL cells [3–5], resembling the immunophenotype of mantle-cell lymphoma. In most CD5(−) MBL cases, clonal B cells display either an unclassifiable or a marginal zone lymphoma- (MZL-) like immunophenotype. However we should take into account that although most marginal zone lymphoma clones are CD5(−), occasionally CD5 is positive.

The frequency of non-CLL-like MBL is significantly lower than that of CLL-like MBL, comprising less than 20% of MBL cases. In the general population studies the prevalence ranges from less than 1% to 2%, depending mainly on the sensitivity of the flow cytometry used [4, 5]. The frequency of non-CLL-like MBLs increases with age. Nieto et al. found a progressively higher frequency of non-CLL-like MBL cases in the general population with increasing age ranging from 0.4% among subjects aged 40–59 years to 5.4% among individuals over 80 years of age with a male predominance [59].

The clinical course of low-count MBL is usually indolent with no evidence of progression to overt lymphoma. A study by Fazi et al., which included only low-count MBL cases, showed that CD5(−) MBL tended to be transient in a significant proportion of cases (3/9) [33]. These results differ from those published in a series of 12 atypical CLL and CD5(−) MBL reevaluated 12 months after the first immunophenotypic analysis [59]. All clones were confirmed and even showed a significant increase in the median concentration of clonal B cells, without however progression to overt lymphoma. Furthermore in the latter study it was shown that a significant proportion of non-CLL-like MBL cases (4/13) showed biclonality.

The biology and clinical significance of clinical non-CLL-like MBL has only recently been investigated. There is only a limited series of patients addressing this issue. Amato et al. reported on 7 cases with CD5(−) non-CLL-like clonal B-cell lymphocytosis with an absolute lymphocyte count ranging from 3,600 to 9,400/*μ*L [60]. The clonal population accounted for 95% to 99% of B cells. The mean age at diagnosis was 72.6 years. Somatic hypermutations of the IGHV gene were found in 6 of 7 cases with different VH gene repertoire from that of CLL. Furthermore, cytogenetic aberrations were found in 5 of 6 cases: 2 cases bearing isochromosome 17q that resulted in loss of p53 and 2 other cases that displayed clones with 7q abnormalities. These latter cases had no evidence of an underlying splenic marginal zone lymphoma. During a follow-up period of 4 to 16 years there was no indication of progression to overt lymphoma. The lymphocytosis was persistent but no progressing.

In 2010 our group described an entity presenting with bone marrow infiltration and blood involvement by CD5(−) lymphocytes of marginal zone origin without any other disease localization, which was named primary bone marrow MZL (PBMMZL) [61]. A total of 23 cases were analyzed; 16 of them presented with lymphocytosis. All patients included in this study had undergone bone marrow evaluation, whole-body CT scan, and gastroscopy. Blood lymphocytes were heterogeneous, consisting mainly of small lymphocytes admixed with medium size lymphocytes with nuclear indentations and monocytoid and villous lymphocytes in various proportions. Blood immunophenotype disclosed a clonal B-cell population with strong expression of CD20 and CD79a and moderate to strong expression of surface light chain; approximately half of the cases also expressed the CD23 and CD11c markers, while all cases were negative for the CD5 antigen. Paraproteinemia was observed in almost half of the cases, mainly of the IgG or IgM type at various levels. Bone marrow was always infiltrated but there was a highly variable degree of infiltration (10–90%, median 25%). The findings of the above study disclosed that CD5(−) MBL displays many similarities with marginal zone lymphomas, mainly the splenic form.

In an attempt to further evaluate these results we performed a comparative study of splenic marginal zone lymphoma (SMZL) and CD5(−) MBL, which disclosed that CD5(−) MBL displays similar features with SMZL regarding morphology of lymphomatous cells, bone marrow infiltration pattern, and immunophenotypic findings [62].

Based on this analysis, we soon came up with a difficulty to characterize and stratify cases with clonal B-cell counts more than 5000/*μ*L, which cannot be characterized as MBL, since there is not currently any recognized entity to include such cases.

**Figure 1:**
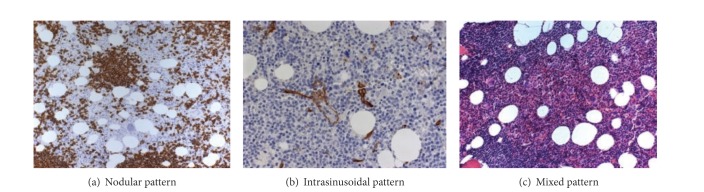
Different patterns of bone marrow infiltration in CD5(−) MBL cases.

In an effort to further investigate this issue we performed an analysis on 44 cases with CD5(−) clonal B cells [63]. 22 of them presented with less than 5000/*μ*L clonal B cells (the median absolute count of clonal B cells was 1123/*μ*L) and could be formally characterized as MBL, while the other 22 cases presented with more than 5000/*μ*L clonal B cells (median count was 6096/*μ*L) and could not be classified under a well-recognized entity. In order to describe these cases, we adopted the term “CD5-monoclonal B-cell lymphoproliferation.” No difference was noticed between the two groups regarding morphology of lymphomatous cells, immunophenotype, pattern, and extent of bone marrow infiltration. Bone marrow evaluation revealed a variable extent of infiltration (median 25%, range 5–80%), while the pattern of infiltration was mixed in the majority of the cases and intrasinusoidal infiltration was evident in one-third of the cases (Figures 1(a), 1(b), and 1(c)). Immunohistochemistry showed a strong expression of CD20 along with negativity for CD5 in all cases, while DBA-44 was positive in approximately half of the cases. Analysis of the IgHV mutation status and of the VH gene usage was performed in 20 cases. Unmutated VH genes were noticed in only 25% of the cases, while the VH4-34∗01 was the most commonly used VH gene. Differences were observed only regarding the clinical course of these two subcategories: in cases with <5000/*μ*L clonal B cells 19/22 (86%) remained stable after a median follow-up time of 27 months, while 2 presented a gradual increase of ALC, without cytopenias or any other features consistent with a lymphoproliferative disorder. In one case MBL regressed a year later. Among MBL cases with >5000/*μ*L clonal B cells after a median follow-up of 48 months (range, 7–154), 10 had stable CBC, 9 had a gradual increase of ALC, 3 progressed, and 2 had resolution of lymphocytosis 6 and 24 months after diagnosis, respectively. Two out of the 3 progressing patients developed cytopenias 57 and 79 months after diagnosis, respectively, while 1 patient developed minimal splenomegaly and pancytopenia 130 months later. These 3 patients were treated with rituximab and achieved complete response. This data showed that CD5-MBL displays many similarities with marginal zone lymphomas and that cases with more than 5000/*μ*L clonal B cells may display a more aggressive clinical course than the typical MBL cases, probably representing a distinct entity. Lymphoplasmacytic lymphoma and IgM-like MGUS are two other entities which may overlap with CD5(−) MBL, at least in a proportion of cases presenting with plasmacytic differentiation and paraproteinemia.

Berger et al. used the term leukemic form in an attempt to characterize lymphomas with MZL features, which could not be classified in any of the subcategories of MZL [64]. Our group had used the term primary bone marrow marginal zone lymphoma in order to describe those cases.

A recent collaborative study on a larger series of patients thoroughly evaluated the clinical aspects, biology, and outcome of 102 non-CLL-like clonal B-cell lymphocytosis cases [65]. The median age at presentation was 70 years with a wide range from less than 40 to more than 90 years, with no sex predilection. Absolute lymphocyte counts ranged from 3,000 to 37,100/*μ*L. Lymphocyte morphology was characterized by heterogeneity, comprised of small lymphocytes, monocytoid-like admixed with variant proportion of villous lymphocytes and lymphocytes with plasmacytoid features. Paraproteinemia was evident in approximately one-third of the cases. The immunophenotype was consistent with a marginal zone derivation since the clonal B cells displayed strong expression of B-cell markers (CD20), moderate to strong expression of surface Ig, and negativity for CD10, with a Matutes score <2. Other markers were positive in a proportion of the cases: CD5 in 18.6%, CD23 in 15.6%, and CD38 in 11.3%. CD79b and FMC-7 were positive in the majority of the cases (>80%). CD49d was positive in all 35 studied cases along with low coexpression of CD38. This was the first published study evaluating the bone marrow histopathology and immunohistochemistry of CBL. In most cases a mixed pattern of infiltration was noticed, mainly interstitial along with intrasinusoidal or nodular. Lymphocytes usually were of small size, while there was a wide variation of the percentage of BM infiltration ranging from less than 10% to more than 70%, with no correlation between the extent of bone marrow infiltration and the absolute number of circulating clonal B cells. Plasmacytic differentiation was presented in some cases. Immunohistochemistry was characterized by expression of CD20 and CD79a, while DBA44 was positive in approximately one-third of the cases. CD5 and CD23 were positive in one and four cases, respectively, but with no coexpression, while cyclin-D1 was always negative.

Cytogenetic analysis revealed an abnormal karyotype in the majority of the cases (~70%), while complex karyotype was found in 23%. The chromosomes most frequently involved included 3, 12, 17, and 7. A high incidence (27%) of aberrations involving chromosome 7 was observed. Del7q which is a typical abnormality in splenic marginal zone lymphoma was detected in 12.5% of MBL cases. Isochromosome 17q was identified in 16.6%. The majority of CBL cases (>70%) carried somatically mutated IGHV genes, with predominance of IGHV4–34 gene. MYD-88 L265P mutation was negative in all of the 45 studied cases. The clinical course varied, with a median follow-up of 5 years; 85 patients remained with isolated lymphocytosis, while 17 cases progressed to an overt lymphoma. Fifteen of them developed splenomegaly, one developed a gastric MALT lymphoma, and one developed diffuse large B-cell lymphoma (DLBCL) of the skin. No difference was found between stable and progressing cases regarding clinical and laboratory characteristics at diagnosis, degree of marrow infiltration, IGHV gene repertoire, and mutational status, while the cytogenetic profiles of the two groups were distinct. Deletions of chromosome 7q were confined to the stable group and complex karyotypes were more frequent in the progressing one. Based on the above study, CBL is closely related to marginal zone lymphomas and in particular to the splenic form: the presence of villous lymphocytes, lymphocytes with plasmacytoid differentiation, intrasinusoidal pattern of bone marrow infiltration, cytogenetic abnormalities of chromosome 7q34, and development of splenomegaly in a significant proportion of cases. According to this study a new term has been initiated which is clonal B-cell lymphocytosis with marginal zone features (MZ-CBL), in order to include cases with CD5(−) clonal B cells irrespective of the absolute number, which fulfill otherwise all the established criteria for MBL diagnosis and proposed to be included in the WHO classification as a provisional entity.

Further studies are required in order to better characterize this entity, identify the relationship with SMZL, and distinguish the majority of cases of MZ-CBL that will remain clinically stable from those destined to progress. Furthermore, no clear recommendations can be made regarding the staging procedures at diagnosis. CT scanning or ultrasonography to exclude nodal and especially splenic enlargement is mandatory, while routine screening for extranodal lymphomas (e.g., gastroscopy) in the absence of specific symptoms is not generally required. Bone marrow examination is mandatory since it provides additional diagnostic information. Since disease progression can occur, often many years after presentation, MZ-CBL cases require long-term follow-up [65].

## 6. Commentary on CD5(−) MBL

On the basis of the aforementioned data the following comments can be made: CD5(−) MBL displays features consistent with a marginal zone origin. The cutoff value of less than 5000/*μ*L CD5(−) clonal B cells cannot be applied in non-CLL-like MBL, since in contrast to CLL, there is not currently a defined entity to include cases with more than 5000/*μ*L CD5(−) clonal blood B cells. The term MZ-CBL can better describe non-CLL-like cases with clonal B-cell lymphocytosis, irrespective of the absolute number of clonal B cells, and it may be regarded as a provisional entity, probably under the name of “primary bone marrow marginal zone lymphoma.” The majority of MZ-CBL present an indolent and stable clinical course. Nevertheless, a proportion of such cases may progress into an overt lymphoma, usually SMZL. Finally, further studies are required in order to better define this entity.

## Figures and Tables

**Table 1:** Prevalence of CLL-type MBL in some of the larger reported series.

Population studied	Median age, Y (range)	*n*	Number of colours	Events ×10^3^	All ages in study, %	CLL-type MBL > 60 y, %	MBL counts (median)
US residential population [9]	53 (40–76)	1926	2	N.R.	0.6	>0.6	88/*μ*L
US blood donors [10]	45 (18–79)	5141	2	N.R.	0.14	0.9	NR
UK hospital outpatients [4]	57 (40–90)	910	4	200	3.5	5.0	0.013 × 10^9^
Italy primary care [5]	74 (65–98)	500	4	200	5.5	5.5	0.114 × 10^9^ (mean)
UK hospital outpatients with normal ALC [11]	74 (62–80)	1520	4	200	5.1	5.1	NR
UK hospital outpatients with lymphocytosis [11]	71 (39–99)	2228	4	200	13.9	NR	NR^)^
Italy residential population [12]	55 (18–102)	1725	5	500	5.2	8.9	0.034 × 10^9^
Spain primary care [3]	62 (40–97)	608	8	5000	14.3	>20	0.17 × 10^9`^
US blood donors [13]	57 (45–91)	2098	6	500	4.8	10.7∗	0.01 × 10^9^(10/*μ*L)
1st-degree relatives of sporadic CLL [14]	62 (18–84)	167	4	300	4.2	15.6	NR
1st-degree relatives of familial CLL [15]	NR	33	2-3	NR	18	NR	NR
1st-degree relatives of familial CLL [16]	47 (23–86)	59	4	NR	13.6	>20	5/*μ*L

NR: not reported.

**Table 2:** Representative studies on the risk of progression of HC-MBL and suggested cutoff values of clonal B cells for predicting the risk of progression.

STUDY	Pts *n*	MEDIAN FUP (years)	Range	CLL/SLL requiring therapy	B-cell count cutoff value
Shim et al., 2007 [9]	*N* = 185 Clinical MBL	6.7	0.2–11.8	1.1% per year	<1900/*μ*L no progression
Shanafelt et al., 2009 [17]	*N* = 459 MBL = 190	1.5	0.3–7.9	1.4% per year	<11000/*μ*L predict better TFS and OS
Rossi et al., 2009 [18]	*N* = 123 Clinical MBL	3.6	NS	4% per year for the first 7 years and then 0%	<1200/*μ*L low risk progression
Scarfo et al., 2012 [19]	*N* = 184 Clinical MBL	3.75	0–306	1.5%	10374 2.4 versus 8.5 5000 1.5 versus 5.2

**Table 3:** Main clinical, cytogenetic, and molecular features of CLL-0, CLL>0, HC-MBL, and LC-MBL.

Characteristics	CLL>0	CLL-0	HC-MBL	LC-MBL
	%			
Annual risk of progression		5.2	1.1	0
Del 13q	50	~40	~40	~30
Trisomy 12	16	~20	~20	~10
Del 11q	18	~5	~5	0
Del 17p	7	2-3	0–3	0
Unmutated IGHV genes	~45	~25	~25	~25
VH1-69	~13	~5	~8	~3
VH4-59/61	<5	~5	<5	~20
BCR stereotypy	~5	~20	~20	~30
NOTCH 1 mutation	>15	13	11	0
